# Resolving Digital Staphylococcal Osteomyelitis Using Bacteriophage—A Case Report

**DOI:** 10.3390/antibiotics7040087

**Published:** 2018-10-02

**Authors:** Randolph Fish, Elizabeth Kutter, Daniel Bryan, Gordon Wheat, Sarah Kuhl

**Affiliations:** 1 PhageBiotics Research Foundation and Grays Harbor Community Hospital, Aberdeen, WA 98520, USA; rcfish4@gmail.com; 2 PhageBiotics Research Foundation, The Evergreen State College, 2700 Evergreen Parkway NW, Olympia, WA 98505, USA; dwbryan@gmail.com; 3 PhageBiotics Research Foundation, Saint Peter Hospital Family Medicine Residency, Olympia, WA 98505, USA; gwheat12@gmail.com; 4 VA Northern California, Muir Road, Martinez, CA 94553, USA; sarahkuhl52@gmail.com

**Keywords:** Bacteriophages, diabetic foot ulcer, osteomyelitis, phage therapy, *Staphylococcus aureus*

## Abstract

Infections involving diabetic foot ulcers (DFU) are a major public health problem and have a substantial negative impact on patient outcomes. Osteomyelitis in an ulcerated foot substantially increases the difficulty of successful treatment. While literature suggests that osteomyelitis in selected patients can sometimes be treated conservatively, with no, or minimal removal of bone, we do not yet have clear treatment guidelines and the standard treatment failure fallback remains amputation. The authors report on the successful treatment, with a long term follow up, of a 63 YO diabetic female with distal phalangeal osteomyelitis using bacteriophage, a form of treatment offering the potential for improved outcomes in this era of escalating antibiotic resistance and the increasingly recognized harms associated with antibiotic therapy.

## 1. Introduction

Diabetic foot infections are a major public-health problem in the US, with high morbidity and long-term costs. We frequently see an impaired wound healing creating disability and amputations, especially in cases complicated by osteomyelitis; this is partly due to antibiotic resistance. The most common infective organism is *Staphylococcus aureus* [1]. Antibiotic treatment of the infection often has poor results and no clear treatment guidelines exist [2]. In one-quarter to one-third of diabetic osteomyelitis cases, the infection is localized in the bones of the toes [3,4].

When treatment for osteomyelitis fails, amputation is the usual option. Prolonged antibiotic-only treatment protocols are sometimes used to try and spare the limb, but are seldom successful [3,4]. Other proposed technologies for osteomyelitis treatment involve topical or local injection of biodegradable materials such as antibiotic-releasing bioactive bone filler materials, antibiotic impregnated collagen sponges or implanted poly-methyl-methacrylate beads [5,6,7,8,9,10]. Dillon injected antibiotics into the tissue surrounding the bone and used a pneumatic boot to reduce edema and increased the local circulation in ischemic feet and legs, reporting a 91.1% ulcer healing success rate and avoiding amputations in one study [11]. Additionally, under evaluation is the protein synthesis inhibitor fusidic acid, used in conjunction with other antibiotics [12]. However, no treatment has been widely adopted that can reliably obviate the need for amputation. This article demonstrates the successful use of *Staphylococcus aureus*-specific bacteriophage injected into a distal toe phalanx and surrounding tissue to resolve the soft tissue infection and osteomyelitis within the phalanx, leading to the complete resolution of the infected ulcer and the osteomyelitis. The patient was seen three years later for a separate problem and we were able to x-ray the original affected toe again, demonstrating that the osteomyelitis was indeed resolved. We have previously reported on a series of six other cases of diabetic toe infections treated with phage after appropriate antibiotics and debridement had failed. (See Discussion).

## 2. Materials and Methods

The phage used is a commercial preparation of staphylococcal phage Sb-1. It was isolated in 1977 for detailed characterization and clinical application from a long-used wound therapy cocktail at the Eliava Institute, located in Tbilisi, Georgia. It is a *Staphylococcus aureus*-specific relative of the phages approved by the FDA for dealing with *Listeria monocytogenes* in ready-to-eat foods. Over a decade ago, Sb-1 was completely sequenced and extensively studied under a special US Department of Health and Human Services Biotechnology Engagement Program (DHHS-BTEP) grant, so we can rule out this phage directly encoding either pathogenicity islands or known transduction mechanisms [13,14]. The phage used here is grown in minimal medium, column purified and sealed in sterile vials in 10-mL aliquots. It was brought into this country as part of a research agreement between the Eliava Institute Phage Production Center and the PhageBiotics Research Foundation, Olympia, Washington, for the purpose of this type of compassionate-use case study.

## 3. Clinical Treatment

The patient was a 63-year-old Caucasian female who presented three months after she developed an ulcer of the distal right second toe. Previous treatment under her primary care provider involved occasional use of topically-applied over-the-counter antibiotic ointment with normal hygiene. Her medical history was positive for type 2 diabetes mellitus, with associated diabetic neuropathy, COPD, hypertension, hyperlipidemia, anxiety disorder, and depression. Physical examination revealed a blood pressure of 160/74 mmHg, pulse of 65 bpm, respirations 20/min and a temperature of 97.4 °F. Pulses measured +1/5 at the dorsalis pedis artery (very weak but palpable) and +3/5 (normal) at the posterior tibial artery. Her legs demonstrated two mm pitting edema. She was morbidly obese, with a BMI of 41.48 and average blood glucose level of 223 mg/dl based on the HgA1c level. Orthopedically, the patient had a contraction deformity of all lesser toes, without bunion deformity. X-rays of the foot revealed osteolysis of the distal phalanx of the right second toe, read as positive for osteomyelitis. 

**Figure 1:**
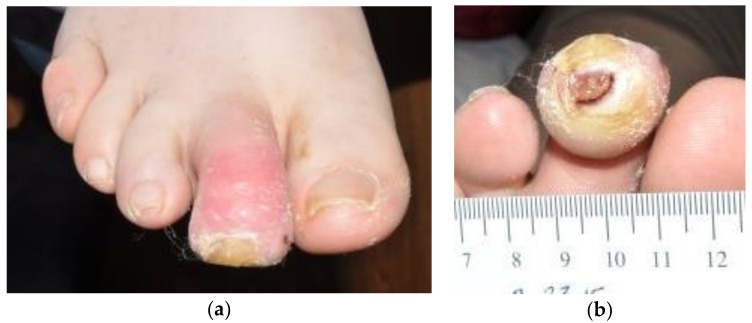
Images of the patient’s ulcer on presentation. (**a**) Top view of foot. (**b**) View of ulcer at the distal tip.

On presentation, she demonstrated an edematous, erythematous, contracted right second mallet toe with ulceration at the distal tip measuring 0.6 × 0.9 × 0.3 cm, probing to the bone (Figure 1). The ulcer bed was primarily fibrin and adipose tissue, with a spotting of red granular tissue. The margins were undermined and the ulcer was without odor. A tissue culture revealed methicillin-sensitive *Staphylococcus aureus,* sensitive to antibiotics except for penicillin.

Treatment options presented to the patient included excision of the distal phalanx along with follow-up antibiotics for at least ten days, or a standard six-week intravenous antibiotic course, together with care of the toe ulcer. She refused both recommendations. After discussion of remaining possibilities, she accepted the use of bacteriophage, as she found it the least objectionable. The treatment consisted of injecting bacteriophage into the soft tissue surrounding the distal phalanx, using a standard injection pattern surrounding the base of the distal phalanx, and inserting directly into the bone as much as possible. The toe was also offloaded with a toe crest and the ulcer treated with standard good wound care. Antibiotics were not used initially.

A week into this treatment, the erythema seemed to be increasing, suggesting the infection was worsening. At this time she also started on levofloxacin 500 mg, to which the bacteria were sensitive. At day seven, we noted no changes in the amount or intensity of the erythema or reduction of edema, suggesting that the antibiotic was not helping, so it was discontinued. No further antibiotics were given.

## 4. Results

**Figure 2:**
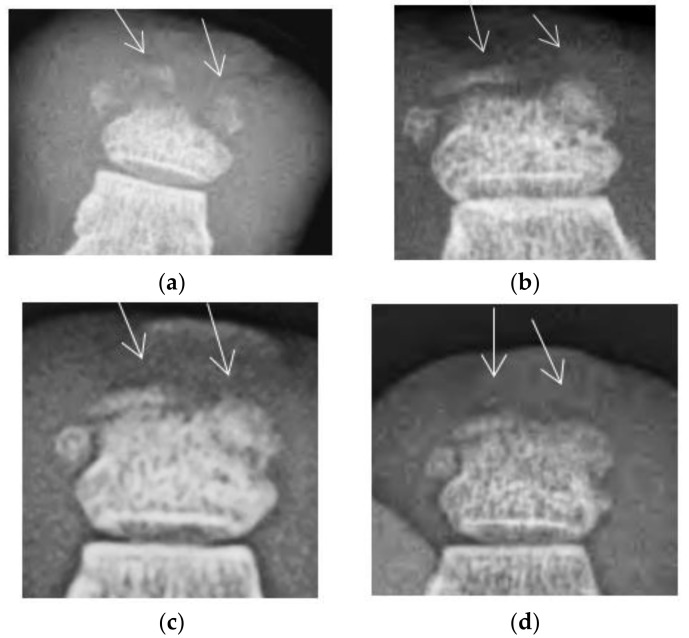
Radiographs of ulcerated toe over the course of treatment showing re-ossification of the toe. (**a**) Radiograph taken on 20 April 2015. (**b**) Radiograph taken on 24 July 2015. (**c**) Radiograph taken on 24 June 2015. (**d**) Radiograph taken on 22 September 2015. Note areas of re-ossification from photo (**a**) to photos (**b**,**c**) and (**d**) (arrows).

The injections began on 23 March 2015 and consisted of 0.7 cc of the highly purified Eliava Institute commercial staphylococcal bacteriophage once weekly for seven weeks, for a total of 4.9 cc. The ulcer healed and the injections were discontinued on 11 May 2015. Serial x-rays demonstrated the re-ossification of the distal phalanx over time (Figure 2). The erythema and edema slowly decreased over the period of treatment, and continued to decrease after the injection treatment was discontinued. The patient was officially discharged on 24 June 2015, but kindly returned for x-rays to follow the progression of the bone healing.

### Long Term Results

**Figure 3:**
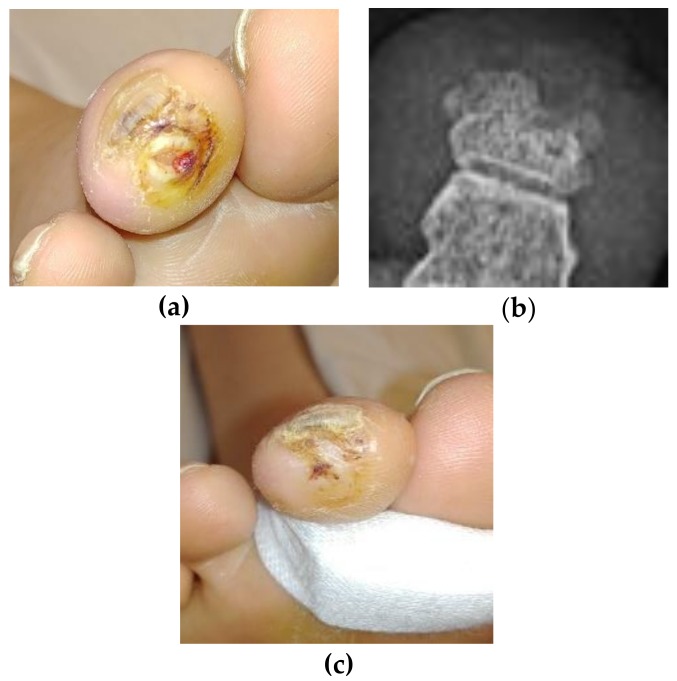
Progression of healing of the ulcer. (**a**) Image of ulcer at the distal tip taken on 30 April 2018. (**b**) Radiograph taken on 30 April 2018. (**c**) Image of closed ulcer at the distal tip taken on 8 May 2018.

Ulcer recurrence in the diabetic population is high, with literature estimating a 40% recurrence rate within one year of the original ulcer healing, and a rate of 60% within three years. Indeed, the best predictor of a recurrent ulcer in a diabetic patient is the occurrence of the first ulcer [15]. In the case of the above patient, she returned to the wound clinic twice in the two years following the resolution of the original ulcer with a single ulcer on a toe of each foot, although on a different toe on the right foot. In April of 2018, she returned a third time with an ulcer of the same toe as the original (Figure 3a), in the same location, although smaller and more superficial. The digital contraction was never resolved and the mallet toe continued weight-bearing beyond physiological limits, causing ulcer recurrence without any sign of recurrent osteomyelitis. This new ulcer was evaluated with x-ray looking for recurrent osteomyelitis. The radiologist read the film as free of osteomyelitis, (Figure 3b) and no further staph phage was used. The ulcer was resolved quickly by using a simple toe crest to stop the pressure on the tip of the toe (Figure 3c). The odd “notch” in the distal phalanx does suggest some original necrosis of the bone and is seen in the earlier films, but we note the re-ossification of the balance of the phalanx when compared to the photos taken three years earlier. 

## 5. Discussion

Here we demonstrate the feasibility of adding bacteriophage to the standard care of diabetic foot ulcers with osteomyelitis, with the successful outcome suggesting the potential for long-term resolution of such infections with phage therapy. We have previously reported the successful treatment of a series of six cases of diabetic toe infection with clinical osteomyelitis [16]. In every case the soft tissue infection and osteomyelitis cleared rapidly. The wounds healed without recurrence indicating successful treatment using this single staph phage, with no further antibiotic therapy. A seventh successful case involved a seriously gangrenous toe salvaged with careful debridement plus the use of this phage to prevent infection.

This case focused on compassionate use of bacteriophage in a patient with Staphylococcal osteomyelitis who refused amputation and/or long-term antibiotics. Only after discussion of the usual treatment options and after the patient refused conventional treatment, bacteriophage treatment was offered as an alternative. After informed consent and following the principals of the Declaration of Helsinki, the treatment was carried out at her next seven weekly visits. Due to the fact that her treatments were carried out in a private physician’s office, no IRB approval was available or necessary. It appears highly unlikely that the successful treatment of this osteomyelitis-complicated ulcer occurred as a result of the one week of levofloxacin early in the treatment, as much longer courses are generally needed for resolution of osteomyelitis if it resolves at all, especially when there is no clinical response to the antibiotic in the first week. The long term follow up also demonstrates that the osteomyelitis was truly resolved, as there was no evidence of disease when she returned three years later, even in the face of a second ulcer in the same location, and with resolution of the ulcer for the previous three years. 

There are important microbiological and other properties of this genus of lytic staphylococcal phages that make them the ideal choice for studying phage treatment of chronic wounds. They have an unusually broad spectrum for various strains of *S. aureus*, and there is very little indication of resistance to these phages even after many years of clinical use across the former Soviet Union. Topical phages can penetrate deeply into infected areas even in the presence of poor circulation, as well as through the thick biofilms common in such chronic wounds. Well-studied phages that have repeatedly been shown to be obligatorily lytic through both classical microbiological techniques and genetic sequencing, such as the staphylococcal phage used in this study, do not pose a significant threat of directly transferring genetic factors that engender virulence or resistance. 

The reintroduction of phage therapy into Western medicine has long been hampered by the difficulty in getting funding for clinical trials due to uncertainties in the regulatory frameworks for such live virus products and some challenging intellectual property issues. The patentability of many obvious phage applications is compromised by the decades-long clinical use of these lytic phages and the very common availability of the phage in the natural world. At least initially, public-private-academic-governmental partnerships appear to be badly needed in order to adequately evaluate the potential for phage therapy. We are working to improve the understanding of the microbiology of diabetic foot infections and the effects of the staph phage on subsequent wound healing by studying the microbial communities with metagenomics data, as discussed by Spichler et al. [17].

Despite innovative thinking and intensive research, comprehensive answers to the accelerating problem of antibiotic resistance still elude us. Further investigations, including controlled clinical trials, are urgently needed to assess the broader potential usefulness of phage as a complementary, narrow-spectrum topical antibacterial, either as an alternative to antibiotics or together with a shorter, more targeted antibiotic treatment. Thus the addition of phage therapy could help prevent antibiotic resistance, treat resistant infections and reduce antibiotic microbiome injury. Wound infections, especially diabetic foot infections with osteomyelitis, offer an excellent opportunity to demonstrate the potential of phage therapy to heal wounds more quickly and effectively while reducing the harms associated with antibiotics.
